# Dr. Stricker on the Hospitals of Italy

**Published:** 1842-12-10

**Authors:** 


### ANALECTA.

*The Hospitals of Italy.* By Dr. Stricker.

*Rome.*—The chief hospital at Rome, for men alone, is the *San Spirito*, near Mount St. Angelo, on both sides of the street. This hospital is capable of containing 2000 patients, and comprehends the lunatic asylum, and the penitentiary for women, *San Michele.* The lunatic asylum, which is still fitted up in the old way, with chains, &c., is not shown to strangers. The medical *clinique* in the hospital is conducted by De Matteis: it consists merely of lectures in a separate theatre. The wards are very large, the kitchen and apothecary's shop well arranged; the latter is provided with a bark-mill. The baths are very elegant, and furnished with materials for vapour baths. The daily ration for non-febrile patients is six ounces of meat and half a *foglietta* of wine. The anatomical lecture-room is very beautiful. The cabinet contains a specimen of a double uterus and vagina; and also wax preparations.

Not far from this, in the Lungara, is the Botanical Garden. The plants are arranged according to the Linnæan system, trees in the higher, and shrubs in the lower part. The walls of the botanical lecture-room are covered with figures of plants. In the *Ospedale degl' Incurabili* on the *Corso*, near the *Piazza del Popolo*, there are 300 beds for surgical patients. The surgical *clinique* is under Titucci, for whose use seven beds are appropriated to male and six to female patients. The spacious slaughter-house is also worthy of notice ; it is situated near the hospital, in the direction of the Tiber. A stream of water flows through it, and it is very clean and judiciously fitted up.

*Florence.*—Two institutions deserve remark in Florence, the one more interesting to the physician, the other to the philosopher. The first is the *Arcispedale di Sta. Maria Nuova*, intended for the practical improvement of the young doctors of the University of Pisa, as this town possesses no hospitals sufficiently large. It is also intended for lectures on every preliminary branch of knowledge, which here, as in the rest of Italy, are gratuitous. The wards of the hospital, which are built in the form of a cross, are extremely clean and orderly ; they contain 2,000 beds. Dr. Betti is the Director, the well-known Bufalini the Professor of the medical *clinique*, and Andreini of the surgical. Sixteen physicians, in all, are attached to the hospital. Besides his morning *clinique*, Bufalini, like the other professors, gives lectures in the hospital. The *gabinetto patologico*, which was founded in 1824, and transferred hither in 1832, is open twice a day. It is well arranged, and particularly rich in diseases of the bones. Many preparations, which would have been damaged if kept in spirit, have been copied in wax. A small botanical garden is connected with the hospital.

The second institution, the *Museo*, which is usually called *Specola*, from the adjoining observatory, is near the Pitti Palace. Antinori is the director, Amici the director of the observatory, Nesti professor of mineralogy and geology, and Mazzi of zoology and zootomy. Since Nobili's death, the chair of physics has been unoccupied. The richest collections are at the service of these professors, the most celebrated of which is the anatomical cabinet of wax figures, which is open to the public daily from 9 to 3.

Contrary to the almost universal usage of Italy, the government takes care that this collection shall progress with the march of science. Thus the germination of cryptogamic plants, represented according to the microscopic investigations of Amici, has lately been added ; and Mazzi is occupied with putting up the shells according to Cuvier's system, and replacing the collection of butterflies, which was much damaged, by a new one. The wax preparations of the zootomical collection are augmented, and the less perfect ones improved, *e. g.* the representation of the development of the chicken in the egg, according to Malpighi.

The physical cabinet, which is particularly interesting, from possessing the tube with which Galileo discovered the satellites of Jupiter, as well as the observatory, contain the newest and most costly instruments.

The hospital of *San Bonifacio*, in the direction of the *Porta San Gallo*, contains in one division an *hospice* for incurable patients, and in the other a well-arranged lunatic asylum, founded under Leopold II.

*The Baths of Lucca* are situated two posts from Lucca, in a romantic valley, between shady mountains. The *Bagni di Villa* are the highest springs, the hottest being the *Bagni caldi*, or *Docce alte*, which are of the temperature of 45° of Reaumur, or 133¼° of Fahrenheit. There are five bathing houses, to which the water is conducted by leaden pipes; the spring *San Bernabo* alone rises near the new and elegant house belonging to it, which contains nine bath rooms and different apparatus for douches, including ascending ones. Its temperature is 32° R. or 104° F. The *Villa*, which is of the same temperature, contains public baths, one for each sex, bathing rooms, and douches. The fifth house is *S. Giovanni*, whose water, as well as that of the *Villa*, is also drunk. Carini is the official physician of the watering-place, and director of the hospital; he comes here only in July and August, Dr. Derè being resident during the rest of the time.

*The Baths of Pisa* are situated at the end of the plain in which Pisa lies, four Italian miles from the town, at the foot of the bare and stony mountain, *San Giuliano.* Medically speaking, they are more efficacious than those of Lucca, but they are destitute of every thing attractive. The only walk is a green planted with trees. The springs are an earthy and saline mineral water, the warm one being of the temperature of 35° R., or $110\frac{3}{4}$° F., the cold one 23° R., or $83\frac{3}{4}$° F. Each of the two bath houses, which stand before the pump-room, is supplied by both springs, and possesses two public baths, one for each sex. They contain, together, thirty-six bathing rooms, which for the most part are provided with douches.

*Genoa* does not belie its epithet of *la superba* in its hospitals, its poor-house, and its university, which are chiefly constructed of marble. The *Albergo dei Poveri*, in front of the *Porta Carbonara*, has beautiful marble stairs; the spacious entrance-hall is adorned with statues of the benefactors of the institution, and is paved with squares of white and black marble, which, in the work-rooms, however, is replaced by bricks in a very bad state. The church is decorated with marble columns, and a bas-relief by Michael Angelo. The institution occupies 1000 women and 800 men. Children are received from their fourth year, and in their fourteenth may resolve whether they will remain there or go out. The girls, when they marry, receive 200 francs as a dowry from the commune. Meat is given three times a week, and wine on Sundays and holidays. There are rooms for the manufacture of wool, silk, linen, cotton, and carpet weaving; a ward for slight cases of illness, the more serious ones being removed to the town; and a school for the children. A prize is given yearly to the best workman.

The great hospital *Pammattone*, in the vicinity of the theatre, is a large square, with arcades, supported by marble pillars, around a court paved with marble. A beautiful marble staircase, covered with statues of benefactors, leads to the wards, which are attended by the sisters of mercy, and where the medical *clinique* is held.

Not far from it stands the *Ospedaletto*, which, in its lower rooms, contains 700 incurable patients of both sexes and all ages; and 300 lunatics up stairs in very dirty wards, the furious ones being together and in chains. For the lunatics are well arranged, but badly situated, an asylum is being built in the marshy tract fronting the *Porta dell' Arco*, near the noisy parade. It consists of a round central building with six wings, in the two lower stories of which are 300 cells; in the third one halls for walking; aud below ground, two dark and padded rooms, eight cells for furious patients, and twelve baths. To prevent accidents the doors are entirely covered with wire lattices, and the stairs up to above a man's height.

In the vicinity of the *Aquasola* walk is the institution for the deaf and dumb, founded in 1818, and now containing thirty-seven pupils, who are instructed in writing, geography, biblical and Roman history, natural philosophy, and religion. The attempt to have the words articulated is not made here. The institution is open to the public from ten to twelve on Wednesday and Friday, while instruction is going on.—*Lond. Med. Gaz.*, Oct. 28, 1842.

